# Under pressure: force resistance measurements in box mites (Actinotrichida, Oribatida)

**DOI:** 10.1186/s12983-019-0325-x

**Published:** 2019-07-04

**Authors:** Sebastian Schmelzle, Nico Blüthgen

**Affiliations:** Department of Biology, Ecological Networks, Technische Universität Darmstadt, Schnittspahnstr. 3, 64287 Darmstadt, Germany

**Keywords:** Euphthiracaroidea, Force measurements, Phthiracaroidea, Predator defense, Ptychoidy, Ptyctima

## Abstract

**Background:**

Mechanical defenses are very common and diverse in prey species, for example in oribatid mites. Here, the probably most complex form of morphological defense is known as ptychoidy, that enables the animals to completely retract the appendages into a secondary cavity and encapsulate themselves. The two groups of ptychoid mites constituting the Ptyctima, i.e. Euphthiracaroidea and Phthiracaroidea, have a hardened cuticle and are well protected against similar sized predators. Euphthiracaroidea additionally feature predator-repelling secretions. Since both taxa evolved within the glandulate group of Oribatida, the question remains why Phthiracaroidea lost this additional protection. In earlier predation bioassays, chemically disarmed specimens of Euphthiracaroidea were cracked by the staphylinid beetle *Othius punctulatus*, whereas equally sized specimens of Phthiracaroidea survived. We thus hypothesized that Phthiracaroidea can withstand significantly more force than Euphthiracaroidea and that the specific body form in each group is key in understanding the loss of chemical defense in Phthiracaroidea. To measure force resistance, we adapted the principle of machines applying compressive forces for very small animals and tested the two ptyctimous taxa as well as the soft-bodied mite *Archegozetes longisetosus*.

**Results:**

Some Phthiracaroidea individuals sustained about 560,000 times their body weight. Their mean resistance was about three times higher, and their mean breaking point in relation to body weight nearly two times higher than Euphthiracaroidea individuals. The breaking point increased with body weight and differed significantly between the two taxa. Across taxa, the absolute force resistance increased sublinearly (with a 0.781 power term) with the animal’s body weight. Force resistance of *A. longisetosus* was inferior in all tests (about half that of Euphthiracaroidea after accounting for body weight). As an important determinant of mechanical resistance in ptychoid mites, the individuals’ cuticle thickness increased sublinearly with body diameter and body mass as well and did not differ significantly between the taxa.

**Conclusion:**

We showed the feasibility of the force resistance measurement method, and our results were consistent with the hypothesis that Phthiracaroidea compensated its lack of chemical secretions by a heavier mechanical resistance based on a different body form and associated build-up of hemolymph pressure (defensive trade-off).

**Electronic supplementary material:**

The online version of this article (10.1186/s12983-019-0325-x) contains supplementary material, which is available to authorized users.

## Background

Heterotroph animals need food to survive, but in many cases, food is not ready to be consumed and many living diets are defended. Thus, food often must initially be made accessible. For example, herbivorous animals must crack the shell of nuts or seeds [14, 30, 31, 42, 62, 74], and predators need to crack the shell of mussels, snails, or other prey such as arthropods [29, 33, 69]. Animals need to search for their respective food sources, and the mechanical resistance of the food or prey increase the consumer’s handling time or even prevent the predation entirely.

Soil habitats are densely packed with a multitude of potential prey from numerous taxa, containing herbivores, detritivores, decomposers, and fungivores, but also other predators [5], representing a particularly strong selection pressure for physical and/or chemical defenses. An important animal group in the soil ecosystem are the Oribatid mites (Actinotrichida). They are speciose and abundant [49], and thus constitute valuable potential prey for predators that naturally also are abundant in soil ([34, 45, 55]; Mollemann and Walter, 2001; [37, 56, 72]). In such scenarios with pronounced predation pressure, oribatid mites developed diverse and effective defensive strategies ([35, 37] and references within), and consequently they are assumed to live in a largely ‘enemy-free space’ [41]. The glandulate Oribatida use chemical defenses that work through the release of secretions by the opisthonotal glands (or ‘oil glands’; [9, 18, 21, 23]) like for example neryl formate, neral, geranial, 2-hydroxy-6-methyl-benzaldehyde (2,6-HMBD; [44]), δ-acaridial [24], and even hydrogen cyanide [10]. Morphological defenses of oribatid mites include a thick, hardened, and in some cases biomineralized cuticle [3, 38, 39], wing-like tecta protecting the legs (pteromorphs; [45, 55]), and erectile setae [36].

**Fig. 1:**
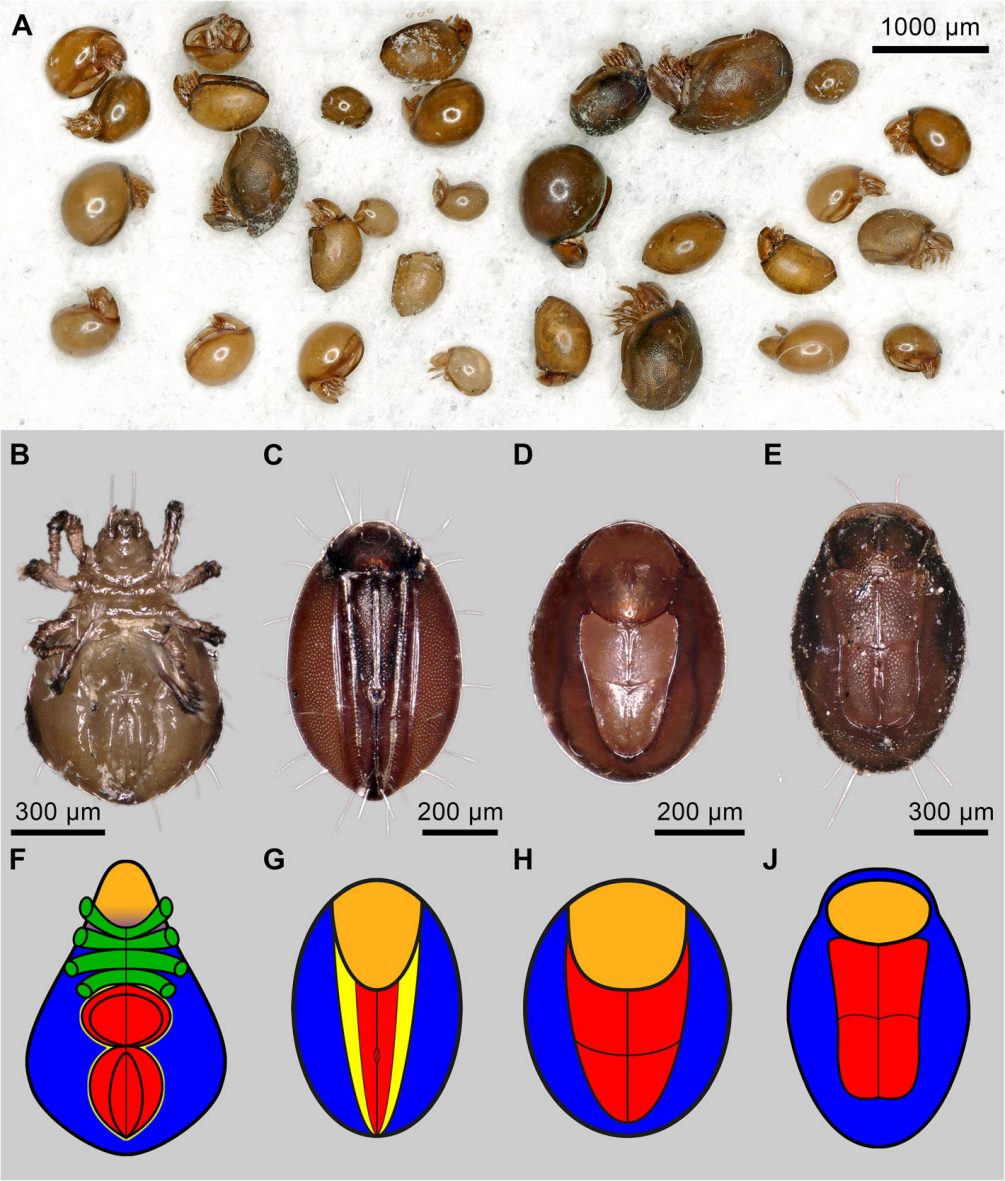
Overview of ptyctimous box mites from a soil and deadwood extraction (**a**) and comparison of the four taxa (**b-j**)**. a** Euphthiracaroidea originated mostly from the deadwood samples, whereas Phthiracaroid mites originated from the leaf litter samples. Photographic (**b**-**e**) and schematic (**f**-**j**) ventral overview of specimens of *Archegozetes longisetosus* (**b**, **f**), Euphthiracaroidea (**c**, **g**), Phthiracaroidea (**d**, **h**) and the genus *Steganacarus* (**e**, **j**) used in the experiment. Blue, notogaster; green, coxisternum and legs (cut off); orange, prodorsum; red, (holo-)ventral plates; yellow, anogenital membrane in *A. longisetosus* (**f**) and plicature plates in Euphthiracaroidea (**g**). The anogenital membrane in Phthiracaroidea (**h**, **j**) is hidden behind the ventral plates and within the idiosoma

**Fig. 2:**
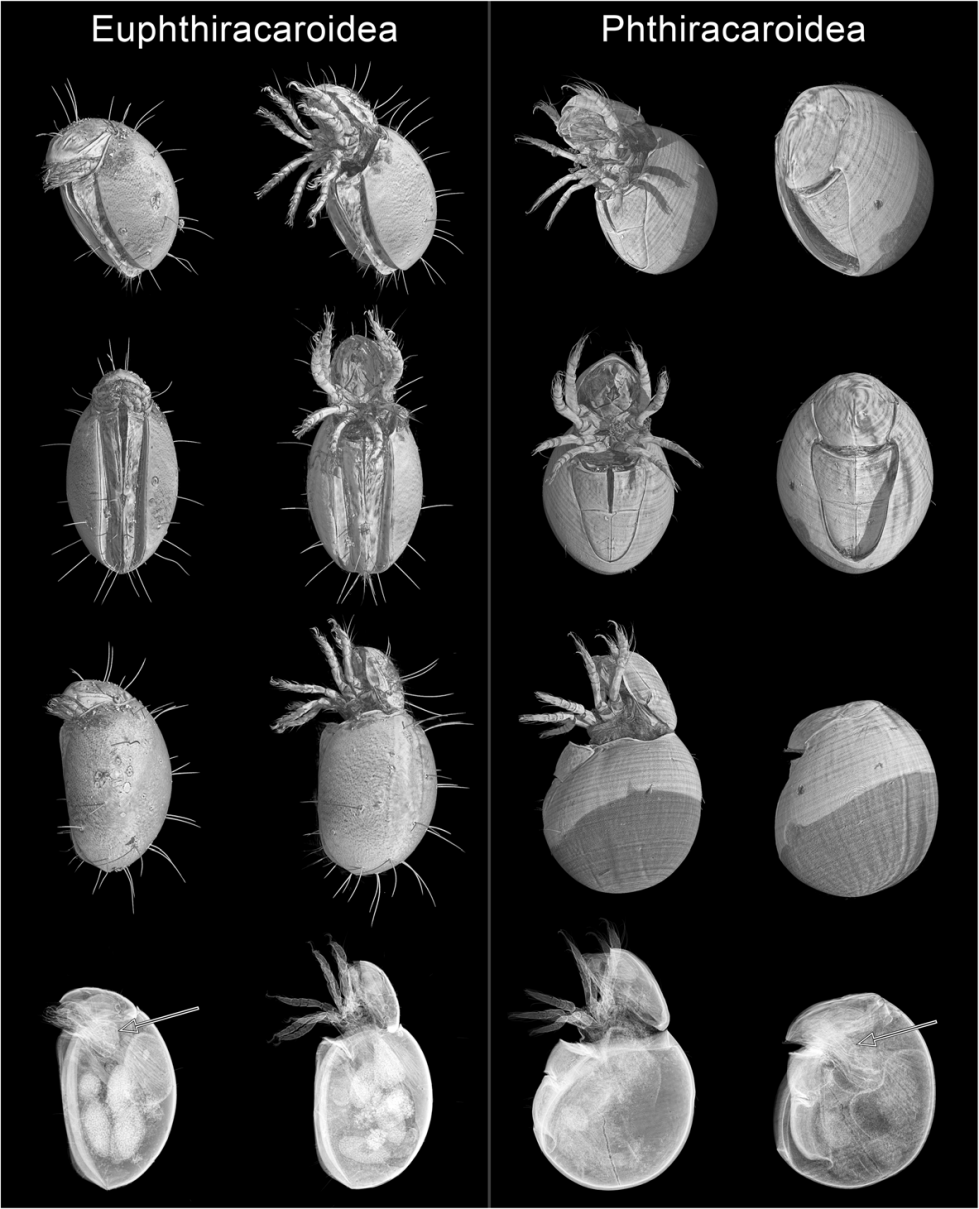
Voxel renderings of specimens of Euphthiracaroidea (left columns) and Phthiracaroidea (right columns) in open (middle columns) and (nearly) encapsulated state (outer columns)**.** First row: antero-ventral view. Second row: ventral view. Third row: lateral view. Fourth row: virtual X-ray image, lateral view. The arrow points towards the legs inside the animal in encapsulated state. Note the muscles attached to the legs responsible for retraction during encapsulation

A particularly complex morphological defensive mechanism in oribatid mites is ptychoidy [45, 47, 54, 55]. It enables the animals to completely retract the appendages into a secondary cavity in the idiosoma and by deflecting the prodorsum encapsulate themselves thereby exhibiting no more soft membrane [47]. Ptychoidy probably evolved three times independently, in the groups Mesoplophoridae, Protoplophoridae (both belonging to the Enarthronota), and in the well-studied Ptyctima (Mixonomata, Fig. 1a; [24, 47, 50–54, 67]). The latter taxon comprises the two groups Euphthiracaroidea and Phthiracaroidea (Figs. 1c-e, g-j and 2) that share a lot of characteristics such as the biomineralized cuticle of similar thickness, but differ in one important morphological key aspect of ptychoidy [54]: the layout of the ventral plates associated with the expansion of idiosomal volume to create space for the appendages during enptychosis (encapsulation of the animals) and the buildup of hemolymph pressure needed for ecptychosis (opening of the animals). To build up pressure, Euphthiracaroidea use lateral compression of the notogaster facilitated by accordion like ventral plates (Figs. 1c, g and 2). whereas Phthiracaroidea (including the genus *Steganacarus*) retract the temporarily unified ventral plates into the notogaster around an anterior fulcrum (Figs. 1d, h, e, j and 2). Both groups are well protected through ptychoidy from attacks of similar sized predators like *Pergamasus septentrionalis* Oudemans [41], *Stratiolaelaps miles* Berlese [24], and small predatory beetles [24]. Euphthiracaroidea, however, additionally feature predator-repelling chemical secretions, that are effective against predators larger than the animals themselves [24]. Since Ptyctima evolved within the glandulate group of Oribatida, it seems likely that Phthiracaroidea secondarily reduced the chemical defense [24]. This hypothesis implies that Phthiracaroidea lost their chemical defense and rely on a hardened notogaster and ptychoidy as defensive systems; so, their effective protection is maintained by an improved mechanical resistance.

The staphylinid beetle *Stenus juno* Fabricius (7 mm long) is neither able to crack Euphthiracaroidea, nor Phthiracaroidea. *Othius punctulatus* Goeze (14 mm long) on the other hand can crack chemically undefended (cf. reducible defense in [20]) euphthiracaroid mites, but not phthiracaroid mites similar in size to Euphthiracaroidea [24]. Ptychoidy thus seems to be less effective against large predators in Euphthiracaroidea than in Phthiracaroidea and we assume that their difference in body form is key for understanding the different armament of defensive systems in the two ptyctimous groups. We hypothesize that equally sized Phthiracaroidea can withstand significantly more force before cracking than Euphthiracaroidea.

Numerous force resistance measurements of prey species have been published [2, 12, 16, 29, 43, 46, 59, 64–66, 69], but to our knowledge, there is none for microarthropods. There are also numerous studies of bite forces that have been determined for many extant – mostly predatory – species [1, 4, 8, 11, 17, 19, 30, 58, 61–63, 68, 71, 73] and also some extinct species [6, 7].

**Fig. 3:**
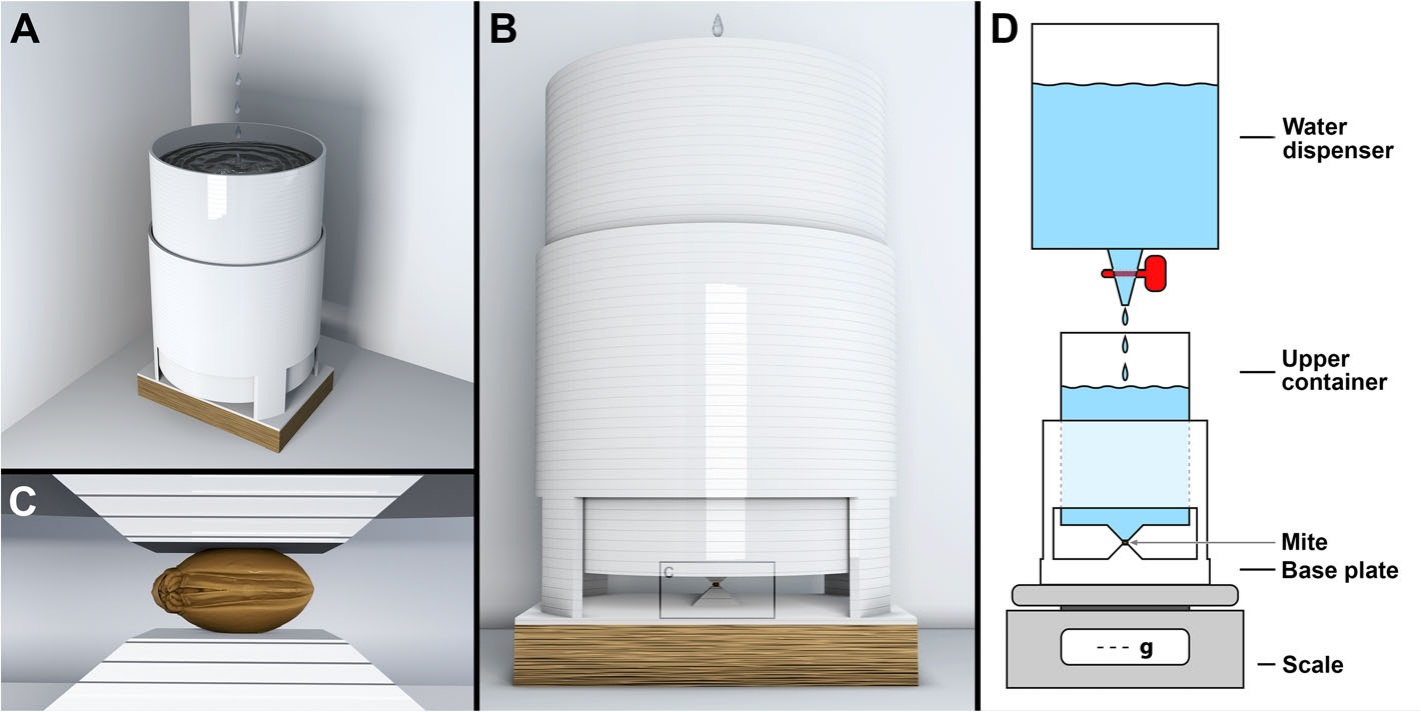
Visualization of the 3D printed test bench consisting of 2 major parts**:** a hollow upper container with a pyramidal extension on its bottom, and a lower test bench with a pyramidal extension for placement of the mites additionally functioning as retainer for the upper container. **a** Overview. **b** Lateral view showing the mite (within inset **c**). **c** Detail showing the mite pinched between the two pyramidal extensions of the upper and lower part. **d** Schematic of the experimental setup

**Fig. 4:**
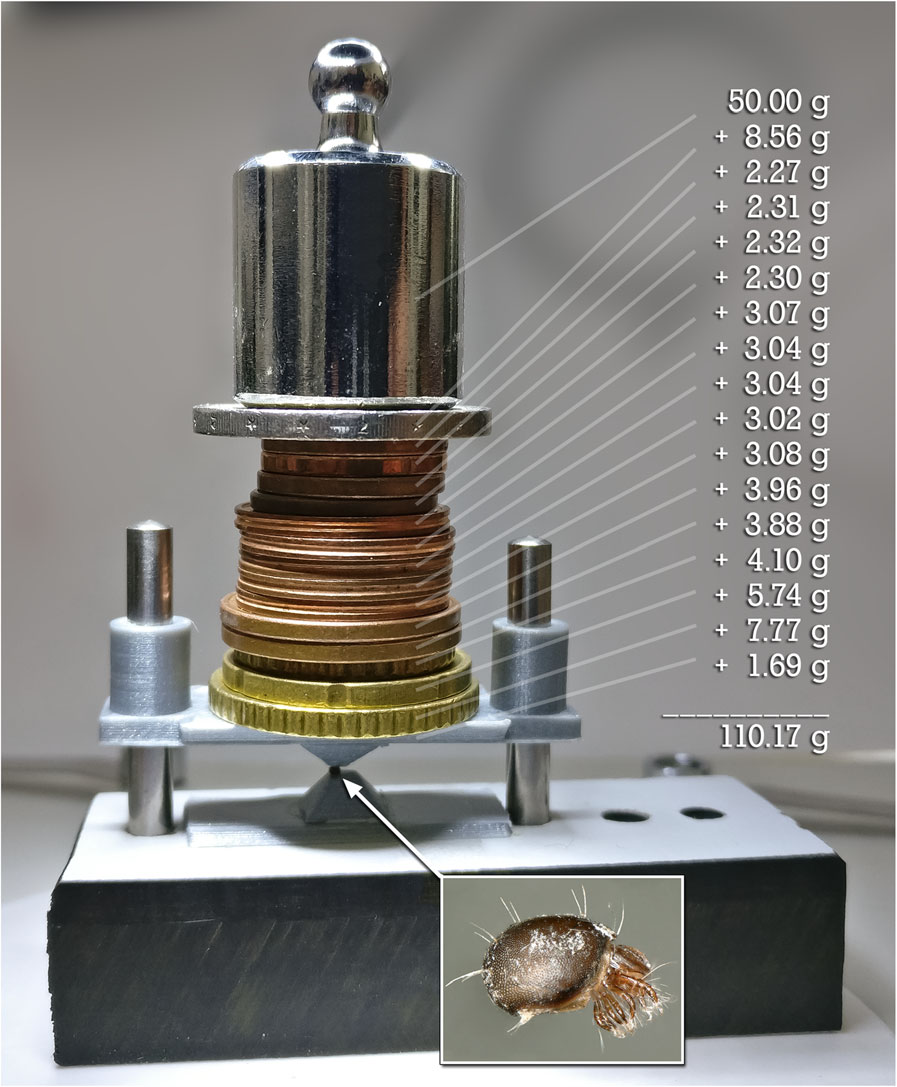
Preliminary study to test the feasibility of the method. The ptychoid mite *Steganacarus magnus* (Phthiracaroidea; another specimen shown in inset) weighing about 420 μg withstood a weight of 110.17 g and thus a force of 1.08 N


We adapted the principle of a Howden compressive testing machine (cf. [64]) to measure force resistance of small species (Figs. 1a and 3; see also Additional file 7: Video S1). Preliminary research with an early version of the test bench proved the feasibility of the method in general (Fig. 4). We then measured force resistance of 125 living ptychoid specimens and additionally 26 living specimens of the soft-bodied mite *Archegozetes longisetosus* Aoki to test our hypothesis, that Phthiracaroidea can sustain significantly more force than Euphthiracaroidea and that their respective distinct body form is key in understanding why.

**Additional file 7: Video S1** Selected videos of the experiment of all taxa (*Archegozetes longisetosus*, Euphthiracaroidea, Phthiracaroidea, *Steganacarus magnus*). Further descriptions are given in the video. (MP4 110503 kb)


## Results

**Fig. 5:**
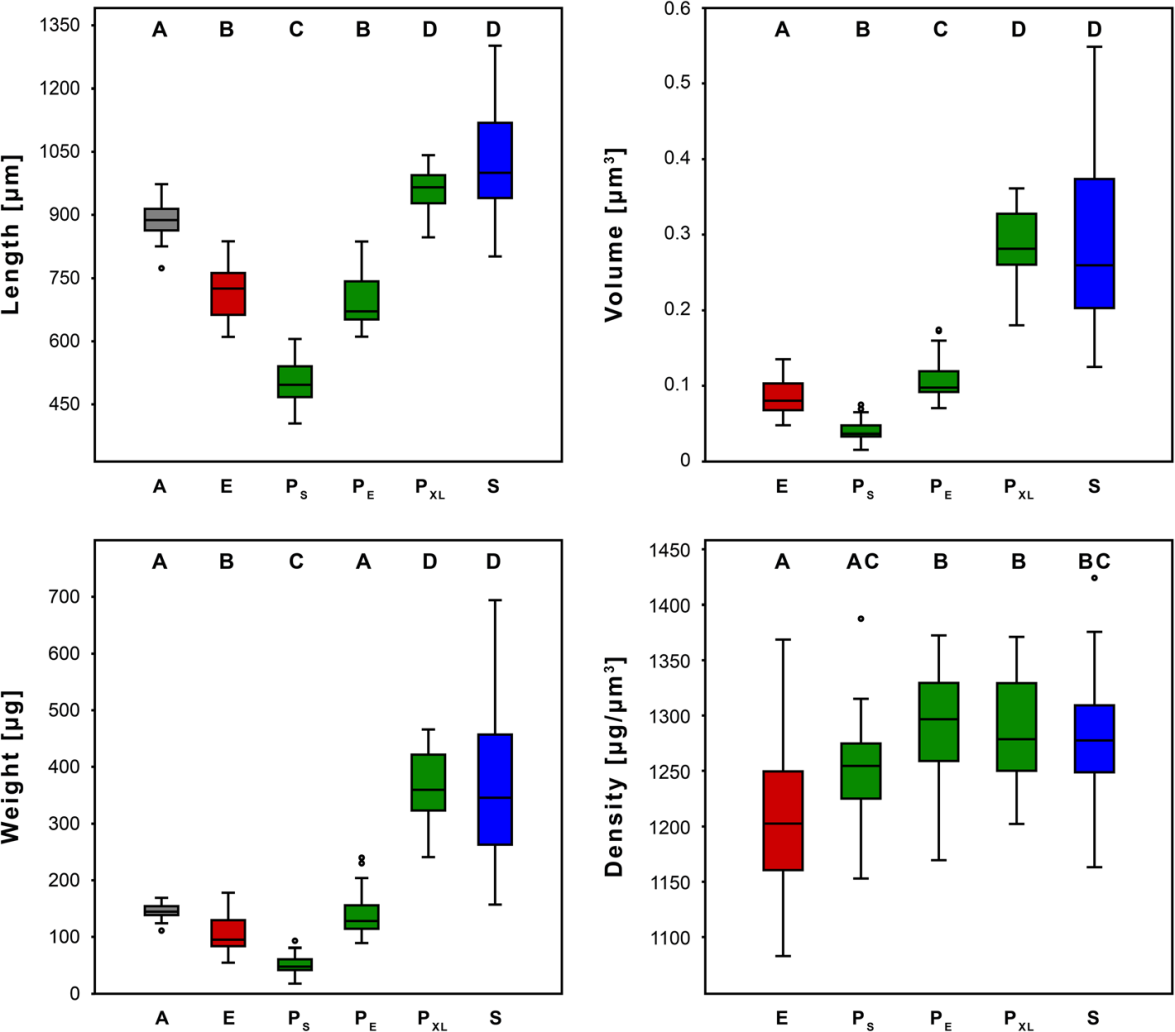
Comparison of different body properties between sample specimens sorted into different species (and in the case of Phthiracaroidea into different body length classes)**.** Upper left: Body length. Upper right: Body volume. Lower left: Body weight. Lower right: Specimen density. A, *Archegozetes longisetosus*; E, Euphthiracaroidea; P, Phthiracaroidea (P_S_, smaller than E; P_E_, same length as E; P_XL_, larger than E); S, *Steganacarus magnus*. Significant differences between groups are indicated by letters above the box plots

**Fig. 6:**
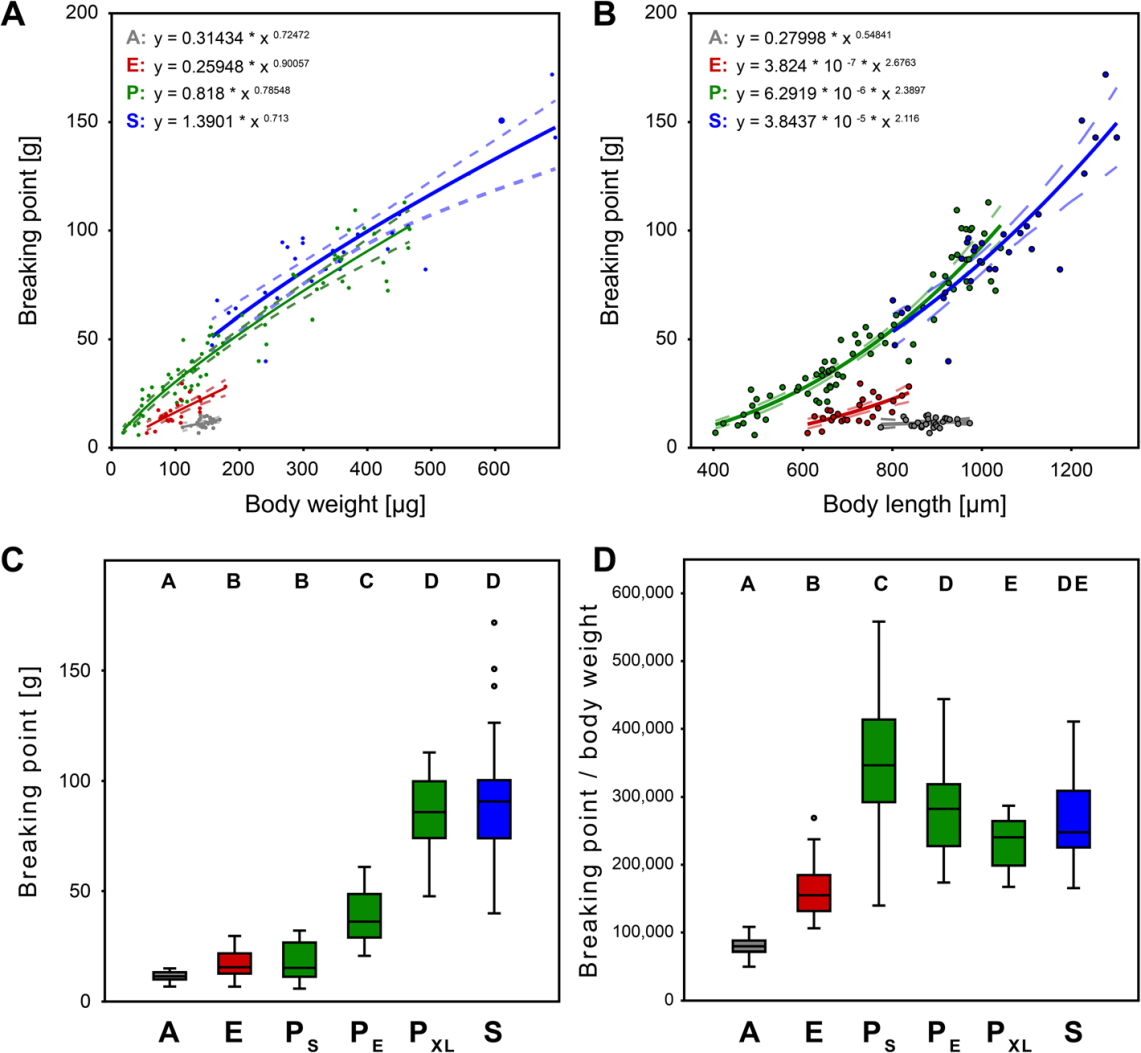
Comparison of breaking point of sample specimens**.** Absolute breaking point per body weight (**a**), absolute breaking point per body length (**b**), and breaking point of sample specimens sorted into different groups (**c**, **d**). **a** Relationship of breaking point [g] and body weight [μg] and non-linear fit (solid lines) with allometric equations and additional 95% confidence intervals (dashed lines) of all taxa. **b** Relationship of breaking point [g] and body length [μm] and non-linear fit (solid lines) with allometric equations and additional 95% confidence intervals (dashed lines) of all taxa. **c** Absolute breaking point. **d** Breaking point per body weight. A, *Archegozetes longisetosus*; E, Euphthiracaroidea; P, Phthiracaroidea (P_S_, smaller than E; P_E_, same length as E; P_XL_, larger than E); S, *Steganacarus magnus*. Significant differences between groups are indicated by letters above the box plots

The variation within taxa was similarly pronounced as the variation between the taxa (Table 1, Figs. 5 and 6). One mite (with a body weight of 689 μg) was able to withstand a maximum weight of 172 g and thus about 250,000 times its body weight (Table 2; Fig. 6c). The maximum recorded relative breaking point, however, was 560,000 times the body weight (the weight of the individual was 48 μg; Table 2; Fig. 6d). The breaking point was significantly different between Phthiracaroidea and Euphthiracaroidea (Tables 2, 3 and 4; Fig. 6; cf. Additional file 1: Table S1, Additional file 3). The mean absolute breaking point of Phthiracaroidea in total (49.3 ± 29.3 g, *N* = 71) was nearly three times that of the Euphthiracaroidea (17.0 ± 6.0 g; *N* = 25). The mean breaking point in relationship to body weight of Phthiracaroidea (281,495 ± 79,135 times their body weight) was nearly two times that of the Euphthiracaroidea (163,076 ± 39,836 times). The mean absolute breaking point of *A. longisetosus* (11.6 ± 2.1 g) was at about 68% that of Euphthiracaroidea, and the relative breaking point to body weight ratio about half that of Euphthiracaroidea (79,972.3 ± 13,539.4 times).

**Table 1:** Body properties of all four groups (Phthiracaroidea without species from the genus *Steganacarus*) including subgroups of Phthiracaroidea categorized by size in comparison to Euphthiracaroidea. All values have been rounded. P_S_, Phthiracaroidea (P) smaller than E; P_E_, P with same respective dimensions as E; P_XL_, P larger than E; sd, standard deviation

	Body length [μm] (mean ± sd; median)	Body weight [μg] (mean ± sd; median)	Body volume [μm^3^] (mean ± sd; median)	Body density [μg/μm^3^] (mean ± sd; median)
*A. longisetosus* (*N* = 26)	774–973 (886 ± 42; 888)	111–169 (145 ± 12; 145)	n.a.	n.a.
Euphthiracaroidea (*N* = 25)	611–837 (716 ± 62.85; 726)	55–178 (104 ± 29.93; 95)	0.048–0.135 (0.086 ± 0.023; 0.08)	1088–1373 (1210 ± 65.62; 1207)
Phthiracaroidea (*N* = 71)	405–1042^b^ (737 ± 184; 690)	18–466^c^ (195 ± 135; 141)	0.016–0.361^c^ (0.151 ± 0.104; 0.106)	1157–1392 (1283 ± 51.37; 1278)
P_S_ (*N* = 17)	405–606 (503 ± 56; 497)	18–93 (52 ± 19; 48)	0.016–0.075^a^ (0.041 ± 0.016; 0.037)	1157–1392 (1254 ± 56; 1259)
P_E_ (*N* = 30)	611–837^b^ (695 ± 60; 671)	89–240^c^ (140 ± 36; 128)	0.07–0.174^c^ (0.107 ± 0.026; 0.098)	1174–1377 (1296 ± 47; 1301)
P_XL_ (*N* = 24)	847–1042 (957 ± 50; 966)	241–466 (365 ± 64; 360)	0.18–0.361 (0.283 ± 0.050; 0.281)	1207–1375 (1288 ± 47; 1283)
*S. magnus* (*N* = 29)	801–1302 (1031 ± 140.45; 1000)	157–694^a^ (373 ± 154.25; 346)	0.125–0.549^b^ (0.291 ± 0.121; 0.26)	1168–1428 (1284 ± 58.69; 1282)

^a^no normal distribution in 1 test: Anderson Darling A or Shapiro-Wilk W)

^b^no normal distribution in 2 tests: Shapiro-Wilk W and Anderson Darling A)

^c^no normal distribution in 3 tests: Shapiro-Wilk W, Anderson Darling A and Jarque-Bera JB)

**Table 2:** Absolute and relative measurement results**.** Phthiracaroidea have been sorted by length. All values have been rounded. A, *Archegozetes longisetosus*; E, Euphthiracaroidea; P, Phthiracaroidea (P_S_, P smaller than E; P_E_, P with same length as E; P_XL_, P larger than E; P_total_, P_S_, P_E_, and P_XL_ combined); S, *Steganacarus magnus*. Numbers in bold indicate the respective lowest and highest values

	A	E	P_total_	P_S_	P_E_	P_XL_	S
*N* (total = 151)	26	25	71	17	30	24	29
Breaking point [g]							
Minimum	6.81	6.78	5.87	**5.87**	20.76	47.65	39.88
Maximum	15.05	29.6	112.95	32.06	60.96	112.95	**171.76**
Mean	11.57	16.98	49.25	18.29	38.6	84.5	93.37
Median	11.44	15.59	40.04	15.3	36.11	85.86	90.69
Standard deviation	2.06	6.01	29.31	8.19	11.29	15.95	29.94
Breaking point [N]							
Minimum	0.067	0.067	0.058	**0.058**	0.204	0.467	0.391
Maximum	0.148	0.29	1.108	0.315	0.598	1.108	**1.685**
Mean	0.114	0.167	0.483	0.179	0.379	0.829	0.916
Median	0.112	0.153	0.393	0.15	0.354	0.843	0.89
Standard deviation	0.02	0.059	0.286	0.08	0.111	0.157	0.294
Breaking point per body weight							
Minimum	**50,074**	106,204	139,762	139,762	173,696	167,292	165,477
Maximum	108,273	269,091	558,333	**558,333**	443,929	287,087	410,788
Mean	79,972.31	163,076.4	281,494.8	353,129.1	279,082	233,769.9	264,568.6
Median	79,649	154,964	270,296	346,818	282,452.5	240,480.5	247,934
Standard deviation	13,539.42	39,835.77	79,134.99	97,656.87	62,903.98	35,478.37	57,106.49

**Table 3:** Analysis of covariance (ANCOVA, type I) describing how the breaking point [N] varies across the four taxa and with body weight (df = degrees of freedom, SS = sum of squares, *F*-value and *p*-value shown; significant effects where *p* < 0.05). Body weight used with power-term b to account for non-linear relationship with N. The interaction term (Taxon × Body weight ^*b*^) describes whether the breaking point increases with body weight at a similar slope across taxa

Predictors (Force)	*df*	*SS*	*F*	*p*
Taxon	3	11.3	503.3	< 2 * 10 ^**−16**^
Body weight^*b*^ [μg]	1	7.2	959.4	< 2 * 10 ^**−16**^
Taxon × Body weight^*b*^	3	0.03	1.1	0.349
Residuals	143	1.1		

*b* = 0.7809425

Numbers in bold indicate significant result

**Table 4:** Results of Kruskal-Wallis test for equal medians and Mann-Whitney pairwise post-hoc test with assumed sequential Bonferroni significance of breaking point between groups. Phthiracaroidea have been sorted by body length. Numbers in bold indicate significant result. A, *Archegozetes longisetosus*; E, Euphthiracaroidea; P, Phthiracaroidea (P_S_, P smaller than E; P_E_, P with same length as E; P_XL_, P larger than E); S, *Steganacarus magnus*

	E	P_S_	P_E_	P_XL_	S
Breaking point [g]					
A	**0.000171**	**0.004812**	1.56*10 ^**−10**^	1.46*10 ^**−9**^	2.19*10 ^**−10**^
E		0.739	5.22*10 ^**−9**^	2.10*10 ^**− 9**^	3.39*10 ^**− 10**^
P_S_			1.18*10 ^**−6**^	7.24*10 ^**−8**^	2.17*10 ^**−8**^
P_E_				1.17*10 ^**−9**^	3.99*10 ^**−10**^
P_XL_					0.396
Kruskal-Wallis test for equal medians: H (chi^2^) = 127.8; Hc (tie corrected) = 127.8; p (same) = 6.82*10^−26^					
Breaking point per body weight					
A	1.09*10 ^**−9**^	4.32*10 ^**−8**^	1.56*10 ^**−10**^	1.46*10 ^**−9**^	2.19*10 ^**− 10**^
E		7.59*10 ^**−7**^	1.57*10 ^**−8**^	1.24*10 ^**−6**^	4.64*10 ^**−8**^
P_S_			**0.003351**	2.74*10 ^**−5**^	**0.000697**
P_E_				**0.005494**	0.4621
P_XL_					0.0784
Kruskal-Wallis test for equal medians: H (chi^2^) = 105.7; Hc (tie corrected) = 105.7; p (same) = 3.36*10^−21^					

The breaking point increased with body weight and differed significantly across taxa (Table 3). Phthiracaroidea and *Steganacarus magnus* withstood significantly more (absolute and relative) force than Euphthiracaroidea (Fig. 6). The non-significant interaction term (Taxon × Body weight ^*b*^) suggests that the increase of breaking point with body weight has a similar slope across taxa. The absolute breaking point increased sublinearly with body weight (proportional to Body weight ^*b*^ hence with power term *b*), where *b* = 0.72 in *A. longisetosus*, *b* = 0.90 in Euphthiracaroidea, *b* = 0.79 in Phthiracaroidea, and *b* = 0.71 in *S. magnus* (Fig. 6a), and on average *b* = 0.7809425 across these taxa (Fig. 6a; Table 3). Furthermore, the sublinearly scaling of the absolute breaking point with body length has more variable power terms *b*, ranging from 0.55 in *A. longisetosus* to 2.69 in Euphthiracaroidea (Fig. 6b).

Similar-sized Phthiracaroidea (P_E_; *N* = 30) withstood significantly more absolute and relative force (mean breaking point of 38.6 ± 11.3 g, 279,082 ± 62,904 times their body weight) than Euphthiracaroidea (Tables 2 and 4; Fig. 6c, d). Specimens of Phthiracaroidea smaller than Euphthiracaroidea (P_S_; *N* = 17) withstood the same absolute force (mean breaking point of 18.3 ± 8.2 g) and withstood significantly more relative force (353,129 ± 97,657 times) than Euphthiracaroidea, similar sized Phthiracaroidea, and larger Phthiracaroidea (P_XL_; *N* = 24; 233,770 ± 35,478 times) (Tables 2 and 4; Fig. 6c, d). In all scenarios the soft bodied mite *A. longisetosus* (*N* = 26) withstood significantly less force than all other groups (mean breaking point of 11.6 ± 2.1 g and an average of 79,972 ± 13,539 times their body weight; Tables 2, 3 and 4; Fig. 6). The breaking point of larger Phthiracaroidea never differed significantly from that of *Steganacarus magnus* (*N* = 29; mean breaking point of 93.4 ± 29.9 g and 264,569 ± 57,106 times; Tables 2 and 4; Fig. 6c, d).

In nine out of the 25 Euphthiracaroidea tested, the prodorsum opened and the legs popped out before the animal was crushed (involuntary ecptychosis; Additional file 7: Video S1 and Additional file 2: Table S2, Additional file 3; cf. Additional file 4: Figure S1). The mean breaking point of these individuals (mean weight of 111.2 ± 24.4 μg) was 12.9 ± 7.1 g and the mean opening weight 11.1 ± 2.8 g. The mean opening weight was thus 62 ± 19% of the mean breaking point in these individuals. The eventual breaking point, however, did not differ significantly (Kruskal-Wallis: *H*(*chi*^*2*^) = 0.013; *p* = 0.91; Additional file 5: Figure S2) between Euphthiracaroidea that showed involuntary ecptychosis (*N* = 9) and those that did not (*N* = 16; mean breaking point of 16.4 ± 5.4 g).

**Fig. 7:**
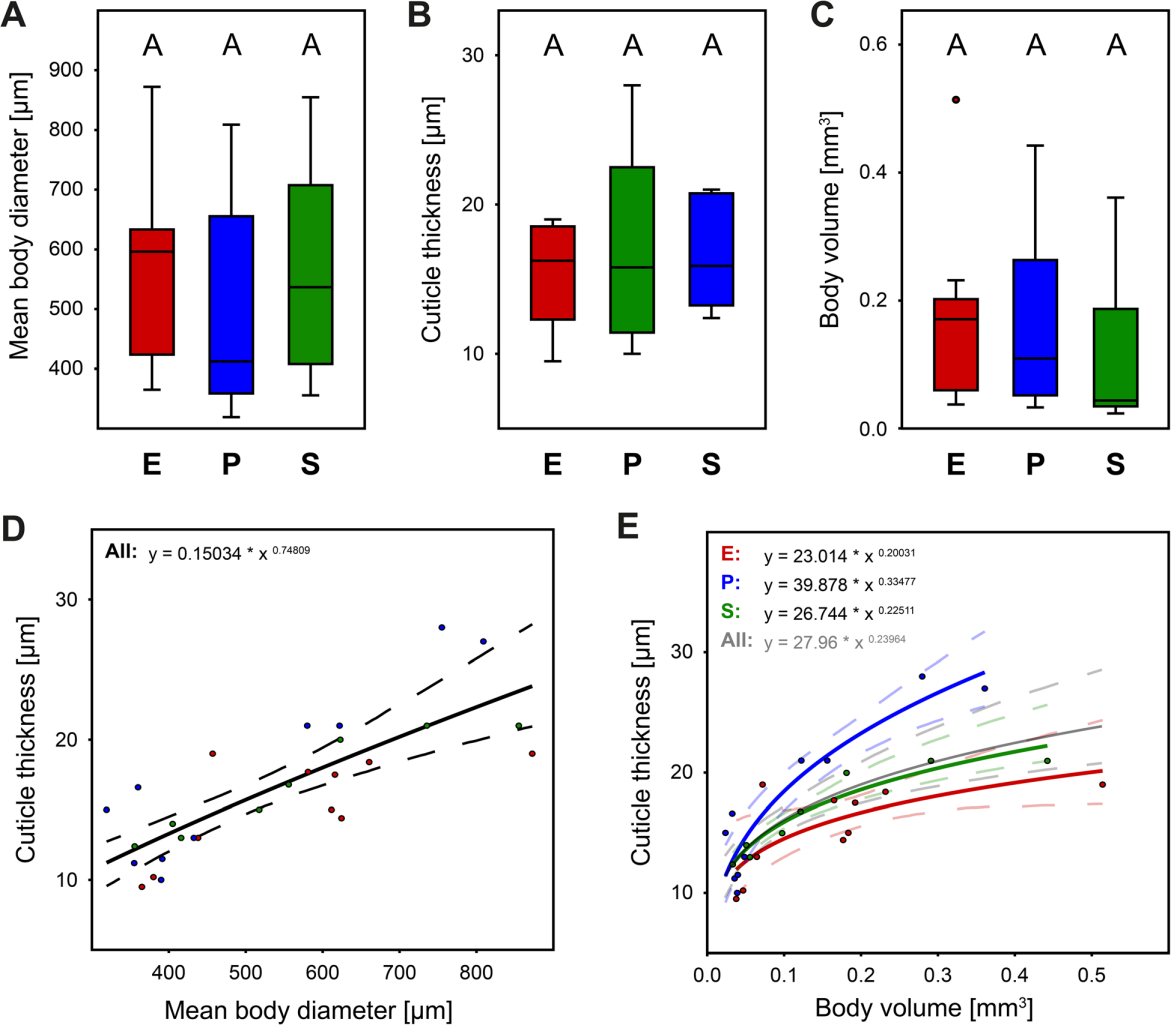
Measurements of cuticle thickness [μm] and body volume [mm^3^] of specimens of Euphthiracaroidea (*N* = 10), Phthiracaroidea (N = 10) and the genus *Steganacarus* (*N* = 8) based on SRμCT data. **a** Comparison of mean body diameter across taxa. **b** Comparison of cuticle thickness across taxa. **c** Comparison of body volume across taxa. The color of the dots corresponds to the respective taxa. **d** Relationship of cuticle thickness [μm] and mean body diameter [μm] and non-linear fit (solid lines) with allometric equation and additional 95% confidence intervals (dashed lines) of all taxa combined. Analysis of the single taxa has been omitted here for clarity. **e** Relationship of cuticle thickness [μm] and body volume [mm^3^] and non-linear fit (solid lines) with allometric equations and additional 95% confidence intervals (dashed lines) of all taxa. All, all taxa combined; E, Euphthiracaroidea; P, Phthiracaroidea; S, *Steganacarus* specimens

Measurements of cuticle thickness and body properties of additional 25 specimen based on SRμCT data showed a high variation (Table 5; Fig. 7). There was no significant difference of body length (*N* = 25; Kruskal-Wallis: *H* = 4.64, *p* = 0.098), mean body diameter (*N* = 25; Kruskal-Wallis: *H* = 1.49, *p* = 0.47; Fig. 7a), body volume (Kruskal-Wallis: *H* = 2.61, *p* = 0.27; Fig. 7c), nor cuticle thickness (Kruskal-Wallis: *H* = 0.46, *p* = 0.79; Fig. 7b) between sample taxa (Table 5). A non-linear fit revealed that cuticle thickness scaled to the 0.75-power with body diameter for all specimen across taxa (Fig. 7d), and that cuticle thickness scaled to the 0.24-power with body volume (*b* = 0.20 for Euphthiracaroidea, *b* = 0.33 for Phthiracaroidea, and *b* = 0.23 for *Steganacarus*; Fig. 7e).

**Table 5:** Cuticle thickness and further body properties of specimens of Euphthiracaroidea, Phthiracaroidea, and specimens of the genus *Steganacarus* (as well as combined total) based on SRμCT data. Rounded values are given as mean (and median) ± standard deviation

	Euphthiracaroidea (*N* = 10)	Phthiracaroidea (*N* = 10)	*Steganacarus sp*. (*N* = 8)	Total (*N* = 28)
Mean length [μm]	849.5 (901) ± 229.82	641.4 (518) ± 216.34	781. 36 (723.4) ± 228.72	755.71 (695.5) ± 234.65
Mean body diameter [μm]	560.6 (596.25) ± 154.22	501.4 (412) ± 177.51	557.93 (536.65) ± 173.26	538.69 (536.65) ± 164.37
Mean volume [mm^3^]	0.168 (0.171) ± 0.140	0.114 (0.044) ± 0.119	0.159 (0.109) ± 0.142	0.146 (0.109) ± 0.131
Mean cuticle thickness *t* [μm]	15.37 (16.25) ± 3.55	17.43 (15.8) ± 6.53	16.65 (15.9) ± 3.59	26.47 (15.8) ± 4.75

## Discussion

**Fig. 8:**
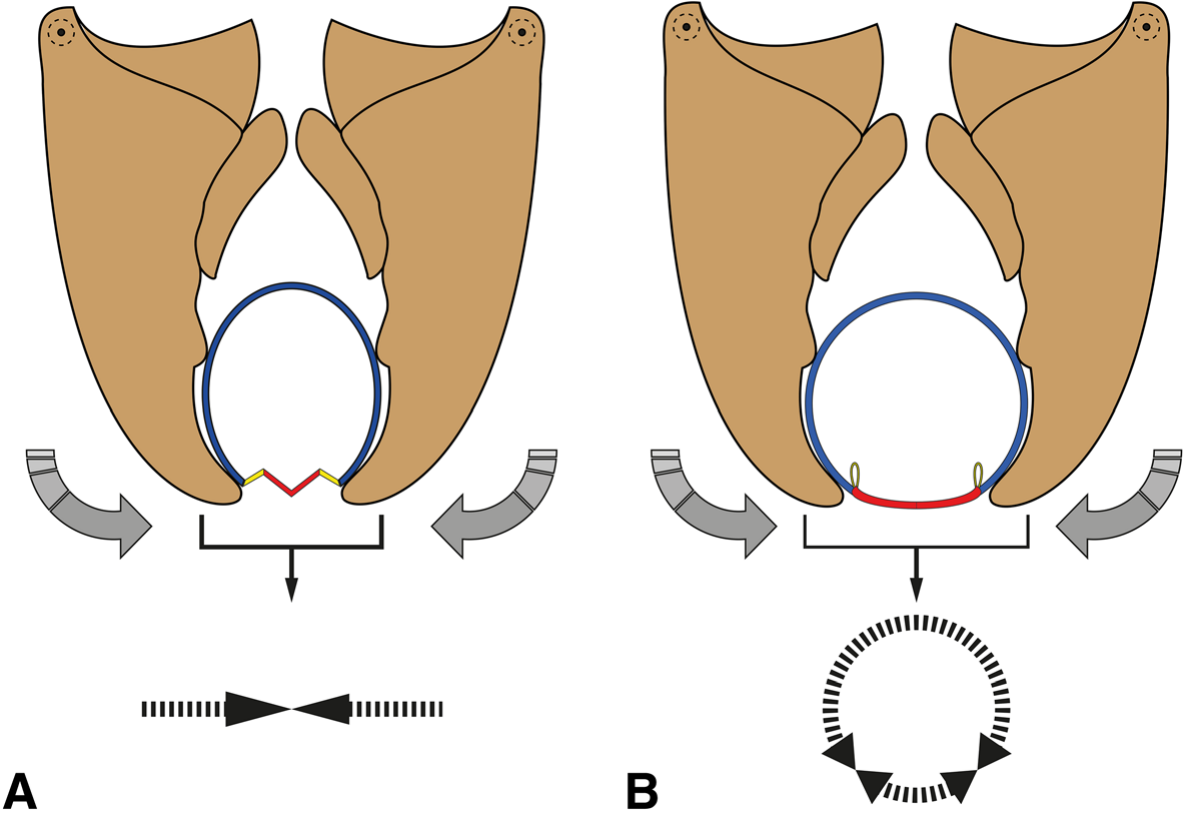
Comparison of body form (represented by cross-sections) of Euphthiracaroidea (**a**) and Phthiracaroidea (**b**) with schematic mandibles of the predatory staphylinid beetle *Othius punctulatus*. The dotted circle indicates the fulcrum of the predator mandibles (in brown). Arrows in upper part indicate the movement (and force) of the closing predator mandibles. The arrows in the lower part indicate the resulting force on the mite body. Blue, notogaster; red, ventral plates; yellow, plicature plates in **a** and anogenital membrane in **b**

We generally confirmed that Phthiracaroidea could sustain significantly more force than Euphthiracaroidea, as expected (Fig. 6). After accounting for the pronounced sublinear increase of resistance with body weight, which was highly variable across and within taxa, the variation across taxa should mirror properties of their cuticle or their shape. Since cuticle thickness was not significantly different (Fig. 7b; Table 5) between both taxa after accounting for body size and since cuticular hardening through biomineralization should be similar in both taxa [38, 39], the main difference across taxa might be explained by their different body forms and structures [54]: Euphthiracaroidea have accordion-like ventral plates (Figs. 1c, g, 2 and 8a) and use lateral compression of the notogaster for hydrostatic pressure compensation. Phthiracaroidea on the other hand retract the temporarily unified ventral plates into the body which in encapsulated state close the ventral notogastral gap leading to a force transmission over the notogastral gap via the ventral plates thereby strengthening the ellipsoid body (Figs. 1d, h, 2 and 8b). This strengthening is lacking in Euphthiracaroidea and force transmission leads to a lateral compression of the ventral plate array and consequently the whole notogaster [54]. As a result, in nine out of 25 cases the forces exerted on some euphthiracaroid specimens led to lateral compression of the notogaster followed by a supposedly involuntary ecptychosis, which, however, did not significantly influence the final breaking point (cf. Additional file 2: Table S2). Although animals in this state would be vulnerable since soft membrane is exposed, we continued until they finally cracked (Additional file 7: Video S1; Additional file 2: Table S2; Additional file 4: Figure S1), which is probably what a larger predator would do when he has sunk his teeth in the prey. Phthiracaroidea have the additional advantage, that the cuticular surface is very smooth, and they are thus prone to slipping out of the grip of predators (see supplementary video S3 in [24]; Additional file 7: Video S1).

The exact mode of failure is unfortunately not observable, since crushing of the mites happens within one frame of the video at normal temporal resolution (60 FPS), but viewing the remains (Additional file 4: Figure S1), we found that especially larger Phthiracaroidea were much more shattered (Additional file 4: Figure S1E, F) than Euphthiracaroidea (Additional file 4: Figure S1A-C). Since Euphthiracaroidea rely on lateral compression of the notogaster the cuticle must retain a certain amount of flexibility, whereas in Phthiracaroidea the cuticle can forgo elastic elements and thus may be more brittle. The highest relative force (about 560,000 times the body weight) was sustained by a rather small phthiracaroid specimen weighing 48 μg at a body length of 484 μm and a body volume of 0.034 μm^3^ (Table 2; Figs. 5 and 6) - roughly corresponding to the weight of the Titanic [15] on an adult human of 80 kg. This might be explained by an increased ratio of cuticle thickness to body volume (Fig. 7e) and smaller body size (Fig. 7d, e) of such small specimens, respectively. Cuticle thickness scaled sublinearly with body diameter and body volume, as did the force resistance (Fig. 6a), hence allometric body size relationships are important for cuticular thickness and mechanical resistance. Accordingly, smaller specimens can resist less absolute, but more relative force per body weight than larger specimens.

Further comparison of the cuticle of ptyctimous mites proves to be difficult since no data is available on the degree of sclerotization, the diameter of microfibers, or fibrous ridges that can influence cuticle strength like was done for other arthropods [12, 66]. Furthermore, testing for example puncture force or tensile strength of the cuticle would require extraction of an isolated piece of cuticle that is as flat as possible, which in these tiny, nearly spherical animals would be very strenuous. Testing the cuticle’s hardness as additional factor though for example nanoindentation, however, could increase comparability and accuracy of the results.

Other oribatid mites without a wide notogastral gap but an integrated ellipsoid idiosomal cuticle instead (like *Oribatella* or *Archipteria*) should be able to sustain even higher forces, but in turn can fall prey to predators because of their often freely accessible and rather unprotected appendages [55]. *A. longisetosus* seems to be an easy and worthwhile prey for predators in regard to force resistance, but it is like Euphthiracaroidea still well defended by predator-repelling chemical secretions [21, 23].

The staphylinid beetle *Othius punctulatus* fed on euphthiracaroid mites in a laboratory experiment in contrast to *Stenus juno*, but not on phthiracaroid mites [24]. This suggests that the bite force of *O. punctulatus* lies between 0.16 and 0.36 N (cf. Table 2). It seems unlikely that oribatid mites constitute the natural diet of this predatory staphylinid beetle since they are probably too small and thus do not fall into the prey size range for a beetle of this size [13, 70], and larger prey should be sufficiently available in soil [5]. Smaller phthiracaroid specimens that can sustain the same forces than Euphthiracaroidea and thus should be ‘crack-able’ are probably of even less interest.

Both groups of Ptyctima are well defended against smaller predators like predatory mites or small staphylinid beetles like *S. juno* [24]. The force needed to crack Euphthiracaroidea, however, would in contrast to similar sized Phthiracaroidea fall well into the bite force range known for predatory beetles: the staphylinid beetle *Ocypus olens* for example can muster a mandibular tip force of 0.195 N (listed as *Staphylinus olens* in [71]) and the highest value recorded by Wheater and Evans [71] was reached by the ground beetle *Abax parallelepipedus* (0.392 N). Larger predators of oribatid mites like poison frogs [48], newts [40], and salamanders [32] should theoretically have higher bite forces, but they rather swallow the mites as a whole. Oribatid mites, however, have been shown to be able to survive the gut passage [60].

Ptychoid mites are particularly resistant compared to other invertebrates: Thaidid snail shells can resist forces about 20,000 times their shell mass [64] and lacustrine gastropods from Lake Tanganyika about 55,000 times their shell mass [69] – about as much as the lowest value which we found for the soft bodied mite *A. longisetosus*. Force resistance measurements in other arthropods such as cockroaches [12] or weevils [65, 66] resulted in failure forces of 1–30 N, and 32–45 N, respectively. Both insect taxa, however, are orders of magnitude larger and heavier than ptychoid oribatid mites.

Under normal conditions, Euphthiracaroidea are well protected by chemistry, whereas Phthiracaroidea lack chemical defense but can withstand significantly higher forces to protect them from potential predators. The evolutionary arms race, however, did not stop at this point and so they too can fall prey to very specialized predators like some species of staphylinid beetles which are roughly in the same size range as their prey (for example so called ‘hole scrapers’; [25–28, 55]).

## Conclusion

Force resistance measurements for small arthropods are feasible with this very simple, low-cost method adapted for small specimens, and showed significant allometric relationships with body mass within taxa as well as systematic differences across taxa. Cuticle thickness also scaled sublinearly with body size and might contribute to variation in resistance, particularly within taxa. Differences in body form, adjustment of body volume and hemolymph pressure were most likely involved in the variation of mechanical resistance across taxa. Our hypothesis that Phthiracaroidea can withstand more force than Euphthiracaroidea was confirmed by the experiment suggesting that Phthiracaroidea afforded to give up chemical secretions because they were well protected mechanically. Hence, they were able to economize a costly defense redundancy. In the future, this method can be used to study force resistance in other potential prey taxa or small food items such as invertebrate eggs and small plant seeds.

## Methods

### Specimens

Ptychoid mites have been extracted (Fig. 1a) from deadwood and leaf litter samples collected near the botanical garden in Darmstadt using Berlese-Tullgren funnels (GPS-locations around N 49.868400, E 8.680519 and N 49.864171, E 8.685044). They were differentiated into the three groups Euphthiracaroidea Jacot (Figs. 1c, g and 2, left columns), Phthiracaroidea Perty (not including the genus *Steganacarus*; Figs. 1d, h and 2, right columns), and *Steganacarus magnus* Nicolet (cf. Fig. 1e, j). Additionally, we used specimens of the parthenogenetic mite *Archegozetes longisetosus* Aoki (Fig. 1b, f) from our own laboratory culture, specifically the lineage Ran originating from a single female collected in Puerto Rico in 1993 [22].

Euphthiracaroidea and Phthiracaroidea have not been identified on a higher taxonomic level (except for specimens of *Steganacarus magnus*), because it was (a) not necessary for testing our hypothesis and (b) because exact identification of phthiracaroid mites on a species level would have required clearing and thus killing or even destroying the mites thereby rendering them unsuitable for testing.

All sample specimens used in the experiment were weighed and measured (Table 1, Additional file 3, Additional file 6: Figure S3). Body weight was measured using a Mettler Toledo XS3DU (max 0.8 g at d = 1 μg, and 3.1 g at d = 10 μg; Mettler-Toledo GmbH, Gießen, Germany). Measurements of body length (l), width (w), and height (h) for ptychoid animals and total length for *A. longisetosus* as well as images of all sample specimens were taken with a Keyence VHX-5000 (KEYENCE DEUTSCHLAND GmbH, Neu-Isenburg, Germany). Since the animals were still alive and active, images of ptychoid animals were mostly taken in encapsulated state from a lateral and ventral view except for a few cases. Then images were taken in partially extended state or from a dorsal view. When possible, length has been measured on lateral and ventral view, and averaged. Images of *A. longisetosus* were always taken from ventral.

In case of ptychoid mites, the measurements were used to calculate the volume using the generalized formula for an ellipsoid, as has been shown to be a good approximation of actual body volume in box mites [54]:

1 $$ V=\frac{4}{3}\times \pi \times \frac{l}{2}\times \frac{w}{2}\times \frac{h}{2} $$

The measurements have then been used to calculate the specific density [μg/μm^3^] of the specimens. Forces were calculated by multiplying the weight on top of the mite comprising the weight of the upper container and the weight of the water by the gravitational acceleration (using the ‘WELMEC’ value for Frankfurt of 9.810476 m/s^2^; [57]).

### Synchrotron X-ray microtomography (SRμCT)

SRμCT data for measurements of cuticle thickness and visualization was originally obtained at the European Synchrotron Radiation Facility (ESRF; experiment SC2127) in Grenoble, France, and the Karlsruhe Research Accelerator KARA at the Karlsruhe Institute of Technology in Karlsruhe, Germany (formerly known as ANKA) within the BMBF-project ASTOR (05K13VTA).

The samples from the ESRF were scanned at beamline ID19 with a beam energy of 20.5 keV, 1500 projections within a 180° rotation, a cooled 14bit CCD camera with a resolution of 2048 * 2048 pixels, and effective pixel sizes of 0.28 and 0.7 μm.

The samples from ANKA were scanned at the TOPO-TOMO beamline with a beam energy of 20 keV, 3000 projections within a 180° rotation (at 300 projections per second), a cooled CCD sensor with a resolution of 2016 * 2016 pixels, and a resulting effective pixel size of 1.22 μm.

Cuticle thickness, body length, height, and width of ten specimens of Euphthiracaroidea, ten specimens of Phthiracaroidea, and eight specimens of the genus *Steganacarus* has been measured with Amira® 5.6.0 (FEI, Munich, Germany). Since *Steganacarus* has a heavily textured, wavy surface measurements of cuticle thickness were taken in the depressions. Voxel renderings were done with VGStudio MAX 3.0 (Volume Graphics GmbH, Heidelberg, Germany).

### Sorting of mites

Phthiracaroid mites have additionally been sorted into the three classes P_S_, P_E_, and P_XL_ regarding the length of the studied Euphthiracaroidea (Table 1, Additional file 1: Tables S1 and S2). P_S_ are individuals of Phthiracaroidea that are smaller than specimens of Euphthiracaroidea, P_E_ have the same dimension, and P_XL_ are larger. Body length was chosen on the assumption that a predator should be able to see and interpret this apparent characteristic and thus treat specimens of both groups with similar size as equally valuable potential prey. The body length groups were determined by the respective size range of Euphthiracaroidea and manually sorted into the three groups.

### Experiments

The test bench consists of two parts (see Fig. 3). The upper part is a hollow container with a pyramidal extension attached to the bottom. To account for the wide range in force resistance we used three upper containers with different dimensions and thus weights. The lower part consists of a flat test bed with a pyramidal extension attached to the top and a retainer in form of a rail guiding the upper container. The whole setup (excluding the water dispenser) is then placed on top of a letter scale (FORACO, maximum 500 g at d = 0.01 g). Since the scale was a ‘no-name’ brand and no further information was obtainable, we tested its accuracy by using steel calibration weights class M2 (± 0.003 g) and found it to be adequate for our experimental design (maximum deviation was + 0.44 g for the 200 g calibration weight and − 0.01 g for the 1 g calibration weight).

Living mites were placed on the pyramidal extension of the base plate. Since euphthiracaroid mites are laterally compressed in comparison to Phthiracaroidea (cf. Figs. 1 and 2) and could thus not reliably be placed on their back for testing, we placed all individual ptychoid mites on the side to keep the results comparable between groups. *A. longisetosus* in contrast was placed on its back, since a placement on its side was impossible. A tiny drop of water was used to keep the mites in place; especially phthiracaroid mites tended to slip out. The base plate with the mite on top was then placed on top of the scale. The scale was tared every time after placing the base plate with the sample specimen on top. Afterwards the upper container was placed on top of the mite with extreme caution. A water dispenser (Brand Titrette; BRAND GMBH + CO KG, Wertheim, Germany) was then used to slowly fill the upper container exerting force via its pyramidal extension on the mite that was placed on the pyramidal extension of the base plate. Once the weight on top of the mites exceeded their resilience, the container completely crushed the mites (Additional file 7: Video S1; Additional file 4: Figure S1) making an afterwards exact species determination impossible. The weight [g] on top of the mite was noted (cf. [64], Additional file 3). Occasionally the prodorsum of Euphthiracaroidea specimens observably popped open and the legs were extruded. The weight on top of the mite at that time was also noted. All tests were filmed (sample specimens pinched between the two pyramidal extensions) using a Panasonic Lumix DMC-GH2 (Panasonic Deutschland, Hamburg, Germany) mounted on a stereomicroscope Zeiss Stemi 2000-C (Carl Zeiss AG, Oberkochen, Germany). Additional images of the experiment were taken using a OnePlus 5 (Shenzhen OnePlus Science & Technology Co., Ltd.; Shenzhen, People’s Republic of China). Renderings were done using Blender 2.79b.

### Statistics

Statistical tests and data visualization have been performed using PAST PAlaeontological STatistics 3.20 and RStudio Version 1.1.456.

To test for significant differences between the taxa and artificially chosen groups within, we made an ANOVA and used a Kruskal-Wallis test for equal medians and a Mann-Whitney pairwise post-hoc test with assumed sequential Bonferroni significance in PAST. The nonlinear fit was tested with the corresponding function in PAST by using the allometric equation function with the ‘zero constant’ variable and 95% confidence interval activated.

Considering all four taxa (without phthiracaroid subgroups), R Studio was used to create a linear model (command *lm*) accounting for respective body properties with

$$ \left(\mathrm{Force}\right)\sim \mathrm{Taxon}\times \left({\mathrm{Body}\ \mathrm{weight}}^{\mathrm{b}}\right) $$

where *b* is the average beta term of the allometric equation based on a non-linear fit of the data (cf. Fig. 6a and the results section).

## Additional files

Additional file 1: **Table S1.** Results of Kruskal-Wallis test for equal medians and Mann-Whitney pairwise post-hoc test with assumed sequential Bonferroni significance of different body properties between groups. (DOCX 19 kb)

Additional file 2: **Table S2.** Body properties of the Euphthiracaroidea specimens where first the prodorsum popped open and the legs were extruded before they were crushed. (DOCX 19 kb)

Additional file 3: Supporting data of the actual experiment and all supporting measurements. (XLSX 26 kb)

Additional file 4: **Figure S1.** Specimens of Euphthiracaroidea (A-C) and Phthiracaroidea (D-F) after the experiment (TIF 6376 kb)

Additional file 5: **Figure S2.** Comparison of eventual absolute breaking point [g] of specimens of Euphthiracaroidea, that showed involuntary ecptychosis (E_inv_; *N* = 9) and those that did not (E; *N* = 16). (TIF 308 kb)

Additional file 6: **Figure S3.** Correlation of body properties of sample specimens sorted into groups (Phthiracaroidea not including the genus Steganacarus). Left column: Body weight per body length. Right column: Body weight per body volume, except first row: voxel rendering of *Archegozetes longisetosus*. Solid line, calculated fit; dotted line, 95% confidence interval; left column, linear fit; right column, allometric equation fit. (TIF 8255 kb)

## Data Availability

All data generated or analyzed during this study are included in this published article [and its supplementary information files, Additional file 3].

## References

[1] Aguirre LF, Herrel A, Van Damme R, Matthysen E (2002). Ecomorphological analysis of trophic niche partitioning in a tropical savannah bat community. Proc Roy Soc Biol Sci Ser B.

[2] Aguirre LF, Herrel A, Van Damme R, Matthysen E (2003). The implications of food hardness for diet in bats. Funct Ecol.

[3] Alberti G, Norton RA, Kasbohm J, Halliday RB, Walter DE, Proctor HC, Ram N, Colloff MJ (2001). Fine structure and mineralization of cuticle in Enarthronota and Lohmannioidea (Acari: Oribatida). Acarology: Proceedings of the 10th International Congress.

[4] Alexander RMcN (1985). The maximum forces exerted by animals. J Exp Biol.

[5] Anderson JM, Vanek J (1975). The enigma of soil animal diversity. Proceedings of the 5th international colloquium on soil zoology held in Prague September 17. - 22. 1973.

[6] Anderson PS, Westneat MW (2007). Feeding mechanics and bite force modelling of the skull of *Dunkleosteus terrelli*, an ancient apex predator. Biol Lett.

[7] Bates KT, Falkingham PL (2012). Estimating maximum bite performance in *Tyrannosaurus rex* using multi-body dynamics. Biol Lett.

[8] Broeckhoven C, Mouton P l FN (2014). Under pressure: morphological and ecological correlates of bite force in the rock-dwelling lizards *Ouroborus cataphractus* and *Karusasaurus polyzonus* (Squamata: Cordylidae). Biol J Linn Soc.

[9] Brückner A, Wehner K, Neis M, Heethoff M (2016). Attack and defense in a gamasid-oribatid mite predator-prey experiment – sclerotization outperforms chemical repellency. Acarologia.

[10] Brückner A, Raspotnig G, Wehner K, Meusinger R, Norton RA, Heethoff M (2017). Storage and release of hydrogen cyanide in a chelicerate (*Oribatula tibialis*). Proc Natl Acad Sci U S A.

[11] Christiansen P, Wroe S (2007). Bite forces and evolutionary adaptations to feeding ecology in carnivores. Ecology.

[12] Clark AJ, Triblehorn JD (2014). Mechanical properties of the cuticles of three cockroach species that differ in their wind-evoked escape behavior. PeerJ.

[13] Cohen JE, Pimm SL, Yodzis P, Saldaña J (1993). Body sizes of animal predators and animal prey in food webs. J Anim Ecol.

[14] Cristol DA, Switzer PV (1999). Avian prey-dropping behavior. II. American crows and walnuts. Behav Ecol.

[15] Dodman F (1952). The observer’s book of ships.

[16] Freeman PW, Lemen CA (2007). Using scissors to quantify hardness of insects: Do bats select for size or hardness?. J Zool.

[17] Goyens J, Dirckx J, Dierick M, van Hoorebeke L, Aerts P (2014). Biomechanical determinants of bite force dimorphism in *Cyclommatus metallifer* stag beetles. J Exp Biol.

[18] Heethoff M (2012). Regeneration of complex defensive oil-gland secretions and its importance for chemical defense in an oribatid mite. J Chem Ecol.

[19] Heethoff M, Norton RA (2009). A new use for synchrotron X-ray microtomography: three-dimensional biomechanical modeling of chelicerate mouthparts and calculation of theoretical bite forces. Invertebr Biol.

[20] Heethoff M, Rall BC (2015). Reducible defence: chemical protection alters the dynamics of predator-prey interactions. Chemoecology.

[21] Heethoff M, Raspotnig G (2012). Expanding the ‘enemy-free space’ for oribatid mites: Evidence for chemical defense of juvenile *Archegozetes longisetosus* against the rove beetle *Stenus juno*. Exp Appl Acarol.

[22] Heethoff M, Laumann M, Bergmann P (2007). Adding to the Reproductive Biology of the Parthenogenetic Oribatid Mite, *Archegozetes longisetosus* (Acari, Oribatida, Trhypochthoniidae). Turk J Zool.

[23] Heethoff M, Koerner L, Norton RA, Raspotnig G (2011). Tasty but protected - first evidence of chemical defense in oribatid mites. J Chem Ecol.

[24] Heethoff M, Brückner A, Schmelzle S, Meusinger R, Dötterl S, Schubert M, Norton RA, Raspotnig G (2018). Life as a fortress – Structure, function, and adaptive values of morphological and chemical defense in the oribatid mite *Euphthiracarus reticulatus* (Actinotrichida). BMC Zool.

[25] Jałoszyński P, Beutel RG (2012). Functional morphology and evolution of specialized mouthparts of Cephenniini (Insecta, Coleoptera, Staphylinidae, Scydmaeninae). Arthropod Struct Dev.

[26] Jałoszyński P, Olszanowski Z (2013). Specialized feeding of *Euconnus pubicollis* (Coleoptera: Staphylinidae: Scydmaeninae) on oribatid mites: Prey preferences and hunting behaviour. Eur J Entomol.

[27] Jałoszyński P, Olszanowski Z (2015). Feeding of *Scydmaenus rufus* (Coleoptera: Staphylinidae: Scydmaeninae) on oribatid and uropodine mites: Prey preferences and hunting behaviour. Eur J Entomol.

[28] Jałoszyński P, Olszanowski Z (2016). Feeding of two species of Scydmaeninae “hole scrapers”, *Cephennium majus* and *C*. *ruthenum* (Coleoptera: Staphylinidae), on oribatid mites. Eur J Entomol.

[29] Lovvorn JR (2004). Relative Foraging Value to Lesser Scaup Ducks of Native and Exotic Clams from San Francisco Bay. Ecology.

[30] Lucas PW, Peters CR, Arrandale SR (1994). Seed-breaking forces exerted by orang-utans with their teeth in captivity and a new technique for estimating forces produced in the wild. Am J Phys Anthropol.

[31] Lundgren JG, Rosentrater KA (2007). The strength of seeds and their destruction by granivorous insects. Arthropod Plant Interact.

[32] Maiorana VC (1978). Behaviour of an unobservable species: diet selection by a salamander. Copeia.

[33] Maron JL (1982). Shell-dropping behavior of Western Seagulls (Larus occidentalis). Auk.

[34] Masuko K (1994). Specialized predation on oribatid mites by two species of the ant genus Myrmecina (Hymenoptera: Formicidae). Psyche (Camb Mass).

[35] Norton RA, Houck M (1994). Evolutionary aspects of Oribatid mite life histories and consequences for the origin of the Astigmata. Mites. Ecological and evolutionary analyses of life-history patterns.

[36] Norton RA, Halliday RB, Walter DE, Proctor HC, Norton RA, Colloff MJ (2001). Systematic relationships of Nothrolohmanniidae and the evolutionary plasticity of body form in Enarthronota (Acari: Oribatida). Acarology: proceedings of the 10th international congress.

[37] Norton RA, Morales-Malacara JB, Behan-Pelletier VM, Ueckermann E, Pérez TM, Estrada-Nenegas EG, Badii M (2007). Holistic acarology and ultimate causes—examples from oribatidmites. Acarology XI: proceedings of the international congress Universidad Nacional Autónoma México.

[38] Norton RA, Behan-Pelletier VM (1991). Calcium carbonate and calcium oxalate as cuticular hardening agents in oribatid mites (Acari: Oribatida). Can J Zool.

[39] Norton RA, Behan-Pelletier VM (1991). Epicuticular calcification in Phyllozetes. Mod Acarology.

[40] Norton RA, MacNamara MC (1976). The common newt (*Notophthalmus viridescens*) as a predator of soil mites in New York. Psyche (Camb Mass).

[41] Peschel K, Norton RA, Scheu S, Maraun M (2006). Do oribatid mites live in enemy-free space? Evidence from feeding experiments with the predatory mite *Pergamasus septentrionalis*. Soil Biol Biochem.

[42] Peters CR (1993). Shell strength and primate seed predation of nontoxic species in eastern and southern Africa. Int J Primatol.

[43] Rajabia H, Darvizeh A, Shafiei A, Eshghi S, Khaheshi A (2014). Experimental andnumericalinvestigations of Otala lactea0s shell–I. Quasi-staticanalysis. J Mech Behav Biomed.

[44] Raspotnig G (2006). Chemical alarm and defence in the oribatid mite *Collohmannia gigantea* (Acari: Oribatida). Exp Appl Acarol.

[45] Riha G (1951). Zur Ökologie der Oribatiden in Kalksteinböden. Zool Jahrb Abt Syst Oekol Geogr Tiere.

[46] Rosin ZM, Kobak J, Lesicki A (2013). Differential shell strength of *Cepaea nemoralis* colour morphs—implications for their anti-predator defence. Naturwissenschaften.

[47] Sanders FH, Norton RA (2004). Anatomy and function of the ptychoid defensive mechanism in the mite *Euphthiracarus cooki* (Acari: Oribatida). J Morphol.

[48] Saporito RA, Donnelly MA, Norton RA, Garraffo HM, Spande TF, Daly JW (2007). Oribatid mites as a major dietary source for alkaloids in poison frogs [published correction appears in proc. Natl. Acad. Sci. USA. 2008 Nov 11;105(45):17586]. Proc Natl Acad Sci U S A.

[49] Schatz H (2004). Diversity and global distribution of oribatid mites (Acari, Oribatida): evaluation of the present state of knowledge. Phytophaga.

[50] Schmelzle S, Helfen L, Norton RA, Heethoff M (2008). The ptychoid defensive mechanism in Euphthiracaroidea (Acari: Oribatida): a comparison of exoskeletal elements. Soil Org.

[51] Schmelzle S, Helfen L, Norton RA, Heethoff M (2009). The ptychoid defensive mechanism in Euphthiracaroidea (Acari: Oribatida): a comparison of muscular elements with functional considerations. Arthropod Struct Dev.

[52] Schmelzle S, Helfen L, Norton RA, Heethoff M (2010). The ptychoid defensive mechanism in *Phthiracarus longulus* (Acari: Oribatida): exoskeletal and muscular elements. Soil Org.

[53] Schmelzle S, Norton RA, Heethoff M (2012). A morphological comparison of two closely related ptychoid oribatid mite species: *Phthiracarus longulus* and *P*. *globosus* (Acari: Oribatida: Phthiracaroidea). Soil Org.

[54] Schmelzle S, Norton RA, Heethoff M (2015). Mechanics of the ptychoid defense mechanism in Ptyctima (Acari, Oribatida): one problem, two solutions. Zool Anz.

[55] Schmid R (1988). Morphologische Anpassungen in einem Räuber-Beute-System: Ameisenkäfer (Scydmaenidae, Staphylinoidea) und gepanzerte Milben (Acari). Zool Jb Syst.

[56] Schneider K, Maraun M (2009). Top-down control of soil microarthropods - evidence from a laboratory experiment. Soil Biol Biochem.

[57] Schwartz R, Lindau A (2002). The new gravity zone concept in Europe for weighing instruments under legal control. Proc. International Conference IMEKO TC3/TC5/TC20 (ed. VDI/VDE-GMA).

[58] Taylor GM (2000). Maximum force production: why are crabs so strong?. Proc R Soc Lond B.

[59] Taylor JRA (2018). Aquatic versus terrestrial crab skeletal support: morphology, mechanics, molting and scaling. J Exp Biol.

[60] Türke M, Lange M, Eisenhauer N (2018). Gut shuttle service: endozoochory of dispersal-limited soil fauna by gastropods. Oecologia.

[61] van der Meij MAA, Bout RG (2004). Scaling of jaw muscle size and maximal bite force in finches. J Exp Biol.

[62] van der Meij MAA, Bout RG (2006). Seed husking time and maximal bite force in finches. J Exp Biol.

[63] van der Meijden A, Langer F, Boistel R, Vagovic P, Heethoff M (2012). Functional morphology and bite performance of raptorial chelicerae of camel spiders (Solifugae). J Exp Biol.

[64] Vermeij GJ, Currey JD (1980). Geographical variation in the strength of thaidid snail shells. Biol Bull.

[65] Wang L-Y, Huang W-S, Tang H-C, Huang L-C, Lin C-P (2018). Too hard to swallow: a secret secondary defence of an aposematic insect. J Exp Biol.

[66] Wang L-Y, Rajabi H, Ghoroubi N, Lin C-P, Gorb SN (2018). Biomechanical strategies underlying the robust body Armour of an aposematic weevil. Front Physiol.

[67] Wauthy G, Leponce M, Banaï N, Sylin G, Lions JC (1998). The backward jump of a box moss mite. Proc R Soc Lond B.

[68] Weihmann T, Reinhardt L, Weißing K, Siebert T, Wipfler B (2015). Fast and powerful: biomechanics and bite forces of the mandibles in the American cockroach Periplaneta americana. PLoS One.

[69] West K, Cohen A, Baron M (1991). Morphology and behavior of crabs and gastropods from Lake Tanganyika, Africa: implications for lacustrine predator-prey coevolution. Evolution.

[70] Wheater CP (1988). Predator-prey size relationships in some Pterostichini (Coleoptera: Carabidae). Coleopt Bull.

[71] Wheater CP, Evans MEG (1989). The mandibular forces and pressures of some predacious Coleoptera. J Insect Physiol.

[72] Wilson EO (2005). Oribatid mite predation by small ants of the genus *Pheidole*. Insect Soc.

[73] Wroe S, McHenry C, Thomason J (2005). Bite club: comparative bite force in big biting mammals and the prediction of predatory behavior in fossil taxa. Proc R Soc Lond B.

[74] Zach R (1979). Shell dropping: decision-making and optimal foraging in northwestern crows. Behaviour.

