# Supplementary material for: Under pressure: force resistance measurements in box mites (Actinotrichida, Oribatida)
Source: Front Zool. 2019 Jul 4;16:24. doi: 10.1186/s12983-019-0325-x (PMC6611053; doi:10.1186/s12983-019-0325-x)
Supplement: Supplementary file 2 — Table S1. Results of Kruskal-Wallis test for equal medians and Mann-Whitney pairwise post-hoc test with assumed sequential Bonferroni significance of different body properties between groups. (DOCX 19 kb) [file 12983_2019_325_MOESM1_ESM.docx]

**Table T1. Results of Kruskal-Wallis test for equal medians and Mann-Whitney pairwise post-hoc test with assumed sequential Bonferroni significance of different body properties between groups.** Numbers in bold indicate significant results. A, *Archegozetes longisetosus*; E, Euphthiracaroidea; P, Phthiracaroidea (P_S_, P smaller than E; P_E_, P with same length as E; P_XL_, P larger than E); S, *Steganacarus magnus*.

| **Body length [µm]** | | | | | |
| --- | --- | --- | --- | --- | --- |
|  | E | P_S_ | P_E_ | P_XL_ | S |
| A | **1.96*10^-9^** | **4.31*10^-8^** | **3.29*10^-10^** | **1.93*10^-5^** | **1.17*10^-5^** |
| E |  | **5.56*10^-8^** | 0.1709 | **2.10*10^-9^** | **8.20*10^-10^** |
| P_S_ |  |  | **1.75*10^-8^** | **7.23*10^-8^** | **2.17*10^-8^** |
| P_E_ |  |  |  | **3.89*10^-10^** | **9.06*10^-11^** |
| P_XL_ |  |  |  |  | 0.0261 |
| Kruskal-Wallis test for equal medians: H (chi^2^) = 127;  Hc (tie corrected) = 127; p (same): 1.05*10^-25^ | | | | | |
|  |  |  |  |  |  |
| **Body volume [µm^3^]** | | | | | |
|  |  | P_S_ | P_E_ | P_XL_ | S |
| E |  | **1.45*10^-6^** | **0.002281** | **2.10*10^-9^** | **5.28*10^-10^** |
| P_S_ |  |  | **1.99*10^-8^** | **7.24*10^-8^** | **2.17*10^-8^** |
| P_E_ |  |  |  | **3.90*10^-10^** | **2.45*10^-10^** |
| P_XL_ |  |  |  |  | 0.4162 |
| Kruskal-Wallis test for equal medians: H (chi^2^) = 103;  Hc (tie corrected) = 103; p (same) = 2.25*10^-21^ | | | | | |
|  |  |  |  |  |  |
| **Body weight [µg]** | | | | | |
|  | E | P_S_ | P_E_ | P_XL_ | S |
| A | **2.43*10^-6^** | **4.24*10^-8^** | 0.08584 | **1.44*10^-9^** | **3.94*10^-10^** |
| E |  | **1.54*10^-6^** | **0.000169** | **2.09*10^-9^** | **4.71*10^-10^** |
| P_S_ |  |  | **1.98*10^-8^** | **7.20*10^-8^** | **2.16*10^-8^** |
| P_E_ |  |  |  | **3.88*10^-10^** | **3.12*10^-10^** |
| P_XL_ |  |  |  |  | 0.5674 |
| Kruskal-Wallis test for equal medians: H (chi^2^) = 125.1;  Hc (tie corrected) = 125.1; p (same) = 2.64*10^-25^ | | | | | |
|  |  |  |  |  |  |
| **Body density [µg/µm^3^]** | | | | | |
|  |  | P_S_ | P_E_ | P_XL_ | S |
| E |  | 0.02579 | **4.45*10^-6^** | **6.07*10^-5^** | **0.00011** |
| P_S_ |  |  | **0.006684** | 0.0354 | 0.05891 |
| P_E_ |  |  |  | 0.5366 | 0.3061 |
| P_XL_ |  |  |  |  | 0.8233 |
| Kruskal-Wallis test for equal medians: H (chi^2^) = 30.29;  Hc (tie corrected) = 30.29; p (same) = 4.28*10^-6^ | | | | | |
