# Supplementary material for: Under pressure: force resistance measurements in box mites (Actinotrichida, Oribatida)
Source: Front Zool. 2019 Jul 4;16:24. doi: 10.1186/s12983-019-0325-x (PMC6611053; doi:10.1186/s12983-019-0325-x)
Supplement: Supplementary file 3 — Table S2. Body properties of the Euphthiracaroidea specimens where first the prodorsum popped open and the legs were extruded before they were crushed. (DOCX 19 kb) [file 12983_2019_325_MOESM2_ESM.docx]

**Table T2. Body properties of the Euphthiracaroidea specimens where the prodorsum popped open and the legs were extruded before they were crushed.** The relative opening weight is regarding the breaking point. All values have been rounded.

| **Individual** | **Weight**  [µg] | **Length**  [µm] | **Volume**  [µm^3^] | **Density**  [µg/ µm^3^] | **Breaking**  **point** [g] | **Opening**  **weight** [g] | **Relative opening weight** |
| --- | --- | --- | --- | --- | --- | --- | --- |
| 8 | 110 | 728 | 0.0876 | 1255.39 | 29.6 | 8.46 | 0.29 |
| 15 | 122 | 757 | 0.0999 | 1220.62 | 25.78 | 15.99 | 0.62 |
| 17 | 87 | 664 | 0.0683 | 1274.07 | 12.66 | 10.58 | 0.84 |
| 23 | 89 | 686.5 | 0.0711 | 1252.18 | 12.29 | 9.5 | 0.77 |
| 25 | 158 | 821.5 | 0.1308 | 1208.04 | 24.05 | 14.61 | 0.61 |
| 28 | 108 | 744 | 0.0993 | 1087.5 | 11.47 | 10.52 | 0.92 |
| 30 | 139 | 802 | 0.1197 | 1161.16 | 21.54 | 13.09 | 0.61 |
| 31 | 95 | 725.5 | 0.0787 | 1206.93 | 12.44 | 9.77 | 0.79 |
| 33 | 93 | 693.5 | 0.0801 | 1160.37 | 12.94 | 7.62 | 0.59 |
|  |  |  |  |  |  |  |  |
| **Summary (n = 9)** |  |  |  |  |  |  |  |
| minimum | 87 | 664 | 0.0711 | 1087.5 | 11.47 | 7.62 | 0.29 |
| maximum | 158 | 821.5 | 0.1308 | 1274.07 | 29.6 | 15.99 | 0.92 |
| median | 108 | 728 | 0.0876 | 1208.04 | 12.94 | 10.52 | 0.62 |
| mean | 111.22 | 735.78 | 0.0928 | 1202.92 | 18.09 | 11.13 | 0.67 |
| standard deviation | 24.40 | 52.18 | 0.0216 | 58.60 | 7.11 | 2.83 | 0.19 |
