# Supplementary figures and images for: Under pressure: force resistance measurements in box mites (Actinotrichida, Oribatida)
Source: Front Zool. 2019 Jul 4;16:24. doi: 10.1186/s12983-019-0325-x (PMC6611053; doi:10.1186/s12983-019-0325-x)

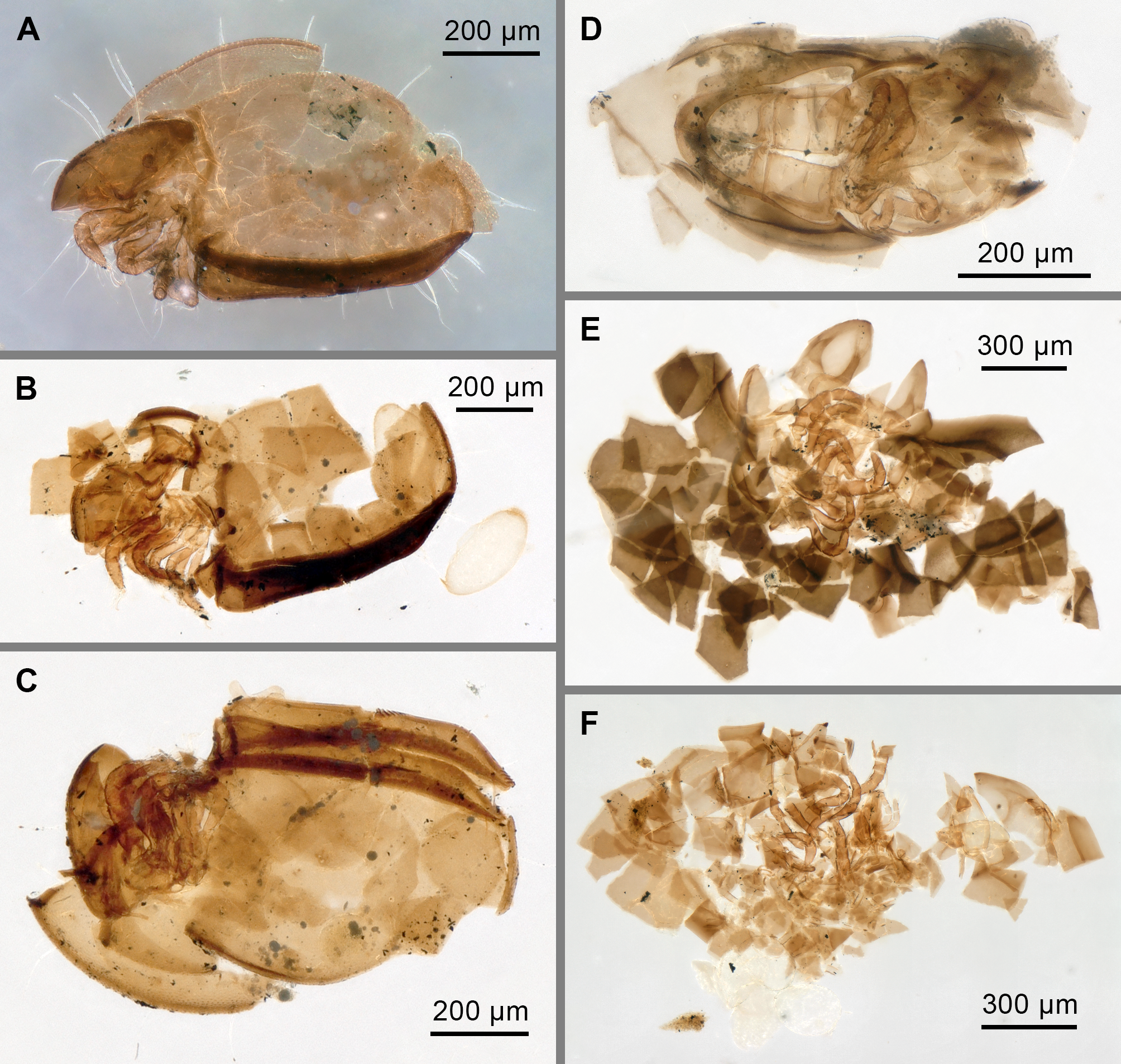

Supplement: Supplementary file 5 — Figure S1. Specimens of Euphthiracaroidea (A-C) and Phthiracaroidea (D-F) after the experiment (TIF 6376 kb) [file 12983_2019_325_MOESM4_ESM.tif]

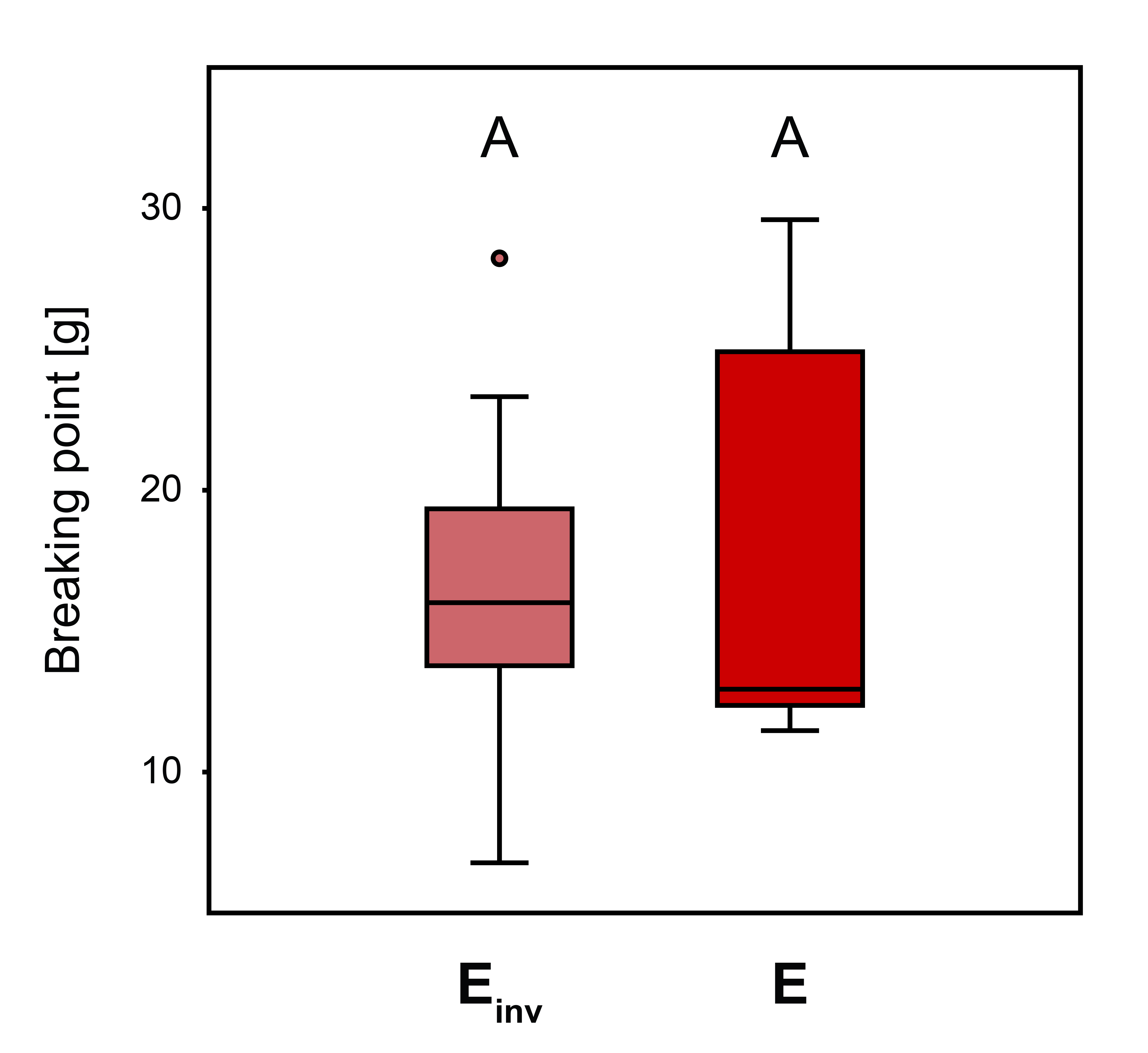

Supplement: Supplementary file 6 — Figure S2. Comparison of eventual absolute breaking point [g] of specimens of Euphthiracaroidea, that showed involuntary ecptychosis (Einv; N = 9) and those that did not (E; N = 16). (TIF 308 kb) [file 12983_2019_325_MOESM5_ESM.tif]
